# Effect of electron contamination on *in vivo* dosimetry for lung block shielding during TBI

**DOI:** 10.1120/jacmp.v17i3.6128

**Published:** 2016-05-08

**Authors:** Ganesh Narayanasamy, Wilbert Cruz, Daniel L. Saenz, Sotirios Stathakis, Niko Papanikolaou, Neil Kirby

**Affiliations:** ^1^ Department of Radiation Oncology University of Texas Health Science Center San Antonio TX USA; ^2^ Department of Radiation Oncology University of Arkansas for Medical Sciences Little Rock AR; ^3^ Landauer Medical Physics Glenwood IL USA

**Keywords:** TBI, lung block, OSLD, dosimetry, electron contamination

## Abstract

Our institution performs *in vivo* verification measurement for each of our total body irradiation (TBI) patients with optically stimulated luminescent dosimeters (OSLD). The lung block verification measurements were commonly higher than expected. The aim of this work is to understand this discrepancy and improve the accuracy of these lung block verification measurements. Initially, the thickness of the lung block was increased to provide adequate lung sparing. Further tests revealed the increase was due to electron contamination dose emanating from the lung block. The thickness of the bolus material covering the OSLD behind the lung block was increased to offset the electron contamination. In addition, the distance from the lung block to the dosimeter was evaluated for its effect on the OSLD reading and found to be clinically insignificant over the range of variability in our clinic. The results show that the improved TBI treatment technique provides for better accuracy of measured dose *in vivo* and consistency of patient setup.

PACS number(s): 87.53.Bn, 87.53.Kn, 87.55.N‐, 87.55.Qr

## I. INTRODUCTION

Total body irradiation (TBI) is a specialized radiotherapy technique used for eradication of residual malignant cells and to cause immunosuppression prior to bone marrow transplant. Uniform irradiation of the whole body is the primary goal of TBI, with the exception of intentionally shielded or boosted areas.^(1)^ Custom fabricated compensators are often required to achieve the required uniformity. This customization along with variations in patient positioning makes these treatments prone to errors. For this reason, *in vivo* dose verification has become essential to ensure the quality of TBI. These measurements are typically made with thermoluminescent dosimeters (TLDs) or optically stimulated luminescent dosimeters (OSLDs), placed at various anatomical sites.^(2)^ This study was motivated by the incidence of higher‐than‐anticipated OSLD readings behind the lung blocks.

## II. MATERIALS AND METHODS

### A. TBI treatment technique

**Figure 1:**
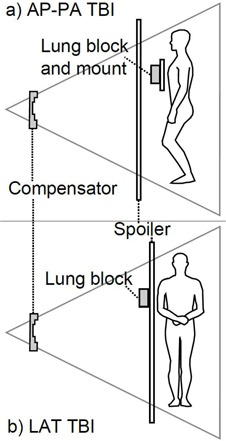
TBI patient treatment geometry showing the lung block and spoiler in front of the patient for (a) AP‐PA and (b) LAT techniques.

Treatments are delivered using a 6 MV photon beam with 40 cm×40 cm field size at the isocenter on a Varian 23EX linear accelerator (Varian Medical Systems, Palo Alto, CA). A typical prescription is 12‐15 Gy in 6 fractions delivered over three days using a bis in die (BID) treatment with the dose rate not exceeding 10‐15 cGy/min at the patient midplane.^3^, ^4^, ^5^ The dose normalization point is set to the patient midplane at the level of the largest separation along the beam direction, typically at the level of umbilicus. Lead compensation filters are fabricated and mounted on the gantry head to compensate for varying body thickness. Custom‐shaped partially transmitting lung blocks are used to lower the lung dose to 25% relative to the full body prescribed fractional dose. Lung blocks are made of Cerrobend alloy (Cerron Metal Products Company, Bellefonte, PA) or Lipowitz metal. These lung blocks are then used for in alternating fractions of treatment, which yields an effective lung total dose that is 62.5% (the average of 100% and 25%) of the prescription dose. In our institution, two TBI treatment techniques are used, both with horizontal beam orientation and a source‐to‐axis distance (SAD) of 340 cm. In the opposing anterior and posterior (AP‐PA) technique, the patient stands upright and in the right–left lateral (LAT) technique, the patient lies on a stretcher. An acrylic beam spoiler is placed in front of the patient to increase the skin dose. In the AP‐PA technique, a spoiler of 1.3 cm thickness is placed 40 cm in front of the patient. In addition, another 1.3 cm thick acrylic is used to mount the lung blocks 20 cm in front of the patient, as shown in Fig. 1(a). In contrast, for the LAT TBI technique, the lung block is mounted outside of the spoiler (0.9 cm thickness), located 16 cm in front of the patient (see Fig. 1(b)).

Entrance dose is measured at the various points of interest, including the head, neck, shoulder, thorax, lung, umbilicus, hip, thigh, knee, and ankle, with screened nanoDot OSLD (Landauer, Chicago, IL). These OSLDs are calibrated for absorbed dose to water in the standard treatment geometry of 100 cm SSD, 10 cm×10 cm field at a the depth of 5 cm (to eliminate electron contamination effects) in a 6 MV clinical beam. Known doses of 0, 12, 25, 50, 100, 150, 200, 250, 300, 400, and 500 cGy were delivered to five OSLDs. Following calibration with four of the OSLDs, a fifth was measured using the newly generated calibration curve to verify the measurements and to examine the single OSLD uncertainty. The fifth verification OSLD agreed within 5.3%, while those specifically in the range of 100‐250 cGy were within 3.1%. The coefficients of variation from the calibration were 0.7% and 1.0% for <100 cGy and 100‐250 cGy, respectively. In order to reduce the uncertainty of entrance skin dose estimation, OSLD measurements were made using a 5 mm bolus.

### B. Determination of Cerrobend 50% dose reduction thickness

Dosimetric measurements were made in the TBI treatment setup to determine the thickness of the Cerrobend lung block required to provide 50% dose reduction. The term 50% dose reduction thickness is used instead of half‐value layer (HVL) since HVL implies halving exposure or air kerma instead of absorbed dose. Two of such layers are employed to limit lung dose to 25% of the open field midline dose. Blocks were made of 2 sizes, 5 cm×5 cm and 10 cm×10 cm, representing the equivalent size of a pediatric and adult lung block, respectively. These two blocks were made with three different thicknesses: 20 mm, 32 mm, and 45 mm. An ADCL‐calibrated PTW Semiflex 31013 ionization chamber (PTW, Freiburg, Germany) with an active volume of 0.3 cc and an ADCL‐calibrated PTW UNIDOS electrometer was used for these measurements. The PTW 31013 ion chamber was placed at 15 mm depth in a 30 cm×30 cm×30 cm solid water phantom centered at 340 cm from the source, which is equivalent to the clinical source‐to‐midline distance. The irradiation was performed with and without the blocks. The readings with block shielding were normalized to the open field readings. The empirical measurement of the 50% dose reduction thickness of the 5 cm×5 cm and 10 cm×10 cm blocks were compared against the theoretical known 50% dose reduction thickness of 16 mm for Cerrobend in a 6 MV beam.^6^, ^7^ This investigation revealed that inadequate thickness of the lung block was being used in TBI at our institution. The measurements were repeated using ion chamber and OSLDs with 5, 10, and 15 mm bolus in identical phantom setup, which leads us to the next part of the investigation.

### C. Variation of bolus thicknesses in OSLD measurements

*In vivo* dosimetry was performed using OSLDs along with a 5 mm bolus affixed to the patient skin. Incidences of higher OSLD reading with the lung block shielding, especially in LAT TBI treatments, prompted an additional set of measurements. The ion chamber measurements described in the previous section, which were used to set the lung block thickness, are performed at depth that would remove electron contamination. For this reason, those measurements do not fully represent *in vivo* verification. To investigate the magnitude of such electron contamination dose, ion chamber and OSLD measurements were performed in a $30\times 30\times 30\;{\text{cm}}^{3}$ solid water phantom centered at 340 cm from source with three different thicknesses of bolus (5, 10, and 15 mm). This was performed with and without lung blocks in both AP‐PA and LAT treatment geometry due to the difference in the spoiler thicknesses and geometry of placement. Results with varying thicknesses of bolus were normalized to the open field readings.

### D. Role of lung block positioning in OSLD measurements

The distance of a lung block from the patient surface may also affect the relative amount of electron contamination. For this reason, the effect of lung block positioning on OSLD measurements was also tested. The 5 cm×5 cm lung block was positioned at points along the central axis (CAX) in four steps of 5 cm increments each, with the three different thicknesses of lung block. OSLD and ion chamber with 5 and 10 mm bolus were affixed in front of the 30 cm×30 cm×30 cm solid water backscatter material centered at a distance of 340 cm from the source. The readings were normalized to the open field readings.

### E. Patient validation

Based on the findings from the Materials & Methods section C above, *in vivo* dose measurements were made using two OSLDs with each lung block (one with 5 mm bolus and another with the new recommended bolus thickness) on four subsequent TBI patients. The two sets of OSLD measurements were compared against the expected skin dose with the lung block shielding which can be estimated for known treatment geometry, based on the fractional dose, depth to midplane, MUs used, and tissue–phantom ratio. The deviation from the expected dose can be compared with mean, standard deviation (SD) and a two‐tailed paired Student's *t*‐test for significance.

## III. RESULTS

Estimation of 50% dose reduction thickness for Cerrobend was made in the TBI treatment geometry using 5 cm×5 cm and 10 cm×10 cm lung blocks using ion chamber at 15 mm depth. 50% dose reduction thickness estimation of both 5 cm×5 cm and 10 cm×10 cm Cerrobend lung blocks yielded a value of 20 mm. In all the subsequent treatments, the thickness of Cerrobend lung blocks were set to 40 mm (two times 50% dose reduction thicknesses), which reduces dose to the intended 25%. The measurements were repeated using ion chamber and OSLDs with 5, 10, and 15 mm bolus thicknesses with and without the lung block shielding. Using the three bolus thicknesses, the ion chamber readings for the open beam were found to be exactly identical to one another. However, the dose measured with lung block shielding using ion chamber and OSLDs was still higher than the expected value for the 5 mm bolus thickness by over 10%.

With a 5 cm×5 cm lung block shielding of two times 50% dose reduction thickness, the ion chamber readings with 5, 10, and 15 mm bolus were found to be 31%, 22%, and 10% of the open beam readings, respectively. The measurements were repeated with OSLDs and found to agree with the ion chamber readings within 6%. In the subsequent treatments, 10 mm bolus thickness was used for OSLD measurements behind the lung block, resulting in measured doses within the acceptable tolerance.

Ion chamber and OSLD measurements using 10 mm bolus thickness were repeated for an open beam and with 40 mm thick Cerrobend lung block shielding. The ion chamber readings matched with the expected value within 1% and the OSLDs agreed to within 3%.

In the last phantom part of the study, the position of the lung block was varied from near the spoiler towards the gantry in four steps of 5 cm increments each. Using a 10 mm bolus material, the readings of the ion chamber in the treatment geometry were 28.1%±0.9% (mean±1 SD) of the open beam measurements. The differences in the ion chamber readings due to lung block position and thicknesses were found to be negligible.

As a part of patient validation, the OSLD readings with 5 mm and 10 mm bolus thicknesses with the lung block shielding were compared against the expected skin dose. The results of the two AP‐PA and the one LAT TBI patients, as well as an irradiation on RANDO phantom (The Phantom Laboratory, Salem, NY) in the lateral TBI geometry, are tabulated in Table 1. Deviation of OSLD reading with 5 mm bolus was outside the 10% tolerance in six out of eight irradiations. Deviation of OSLD reading with 10 mm bolus was within tolerance in all the eight irradiations, barring once when the OSLD fell off Patient #1 during treatment and was not read. The mean±1 SD of the readings dropped from 22.5%±0.2% with 5 mm bolus to 6.4%±0.03% with 10 mm bolus. OSLD reading with 10 mm bolus has a better agreement with the expected entrance dose than that with 5 mm bolus, with the lone exception of PA beam in Patient #3 when the OSLD was away from the center of the blocked region. The differences from the two sets of readings were found to be statistically significant in a two‐tailed paired Student's *t*‐test with a p‐value <0.03.

**Table 1:** Percentage deviation of OSLD reading with 5 mm and 10 mm bolus from the expected entrance dose with lung block shielding in two LAT TBI and two AP‐PA TBI patients. Entrance dose reading of the right lateral field in Patient #1 was not obtained with 10 mm bolus due to OSLD falling off the patient during treatment.

*Patient #*	*Beam Orientation*	*Percent Deviation of OSLD Reading with 5 mm Bolus*	*Percent Deviation of OSLD Reading with 10 mm Bolus*
1^a^	Right Lateral	15.9 %	6.6%
	Left Lateral	25.5%	8.5%
2	Right Lateral	51.6%	7.9%
	Left Lateral	44.5%	8.7%
3	AP	21.2%	8.0%
	PA	10.4%	6.8%
4	AP	29.7%	7.7%
	PA	15.2%	7.5%
Mean difference		22.5%	6.4%
SD		0.19%	0.03%

a Data from a RANDO phantom irradiation in the lateral TBI patient treatment geometry.

## IV. DISCUSSION

The entrance skin dose with the lung block shielding depends on multiple factors — block and bolus thickness. The LAT TBI treatment geometry places less material between the lung block and the patient than the AP‐PA TBI setup. The spoiler used in LAT treatments was 4 mm thinner than in AP‐PA treatment. This produces a higher contribution from electron contamination and higher OSLD readings with 5 mm bolus for LAT TBI treatments than for AP‐PA treatments. As shown in Table 1, the higher dose readings behind the lung block are offset by using a bolus of 10 mm thickness. In the subsequent AP‐PA and LAT TBI treatments, the *in vivo* measured doses were within the clinically accepted tolerance of 10% from the expected value. Although placement of lung block showed negligible effect in the OSLD reading, care should be taken to block the lung completely by repositioning the lung block at the same position during treatment.

TLDs are also commonly used for *in vivo* dosimetry of TBI and we have seen similar consistency with the use of OSLDs.^(8)^ Although the report mentions the technical challenges with TLD calibration for lung block shielding due to change in the radiation field composition, it fell short on identifying the nature of spectrum changes. Our hypothesis of electron contamination emanating from the lung block was subsequently verified with ion chamber and OSLD readings with varying bolus thicknesses. Care should be taken to ensure appropriate placement of OSLD on the patient near the center of the blocked region. A more recent TLD‐based *in vivo* dosimetry study on 20 TBI patients was reported in literature, although without lung block shielding.^(9)^

A study on lung block positioning found deviation <5 mm and <10 mm were acceptable along the horizontal and vertical directions, respectively.^(10)^ We had included the displacement of the lung block shield along the CAX in this study, which was a more common occurrence. As a part of our institutional policy, the lung block positioning was verified using imaging prior to treatment delivery.

The individual contributions of photon and electron contaminations were not addressed in this manuscript. This study not only helped in closing the gap in our understanding, but also improved the clinical work flow of *in vivo* dosimetry of TBI.

## V. CONCLUSIONS

Electron contamination from lung blocks caused our *in vivo* OSLD measurements to be higher than expected. Artificially high readings may cause one to make unnecessary compensation filter or lung block modifications. This was remedied by increasing our OSLD bolus thickness from 5 mm to 10 mm. The new *in vivo* measurements made in both AP‐PA and LAT TBI treatment techniques increase confidence in the dosimetry and delivery of TBI, ensuring that an appropriate dose was delivered to the patient.

## COPYRIGHT

This work is licensed under a Creative Commons Attribution 4.0 International License.
